# Adaptive mutation F772S-enhanced p7-NS4A cooperation facilitates the assembly and release of hepatitis C virus and is associated with lipid droplet enlargement

**DOI:** 10.1038/s41426-018-0140-z

**Published:** 2018-08-08

**Authors:** Xiaobing Duan, Muhammad Ikram Anwar, Zhanxue Xu, Ling Ma, Guosheng Yuan, Yiyi Chen, Xi Liu, Jinyu Xia, Yuanping Zhou, Yi-Ping Li

**Affiliations:** 1 Institute of Human Virology and Zhongshan School of Medicine, Sun Yat-Sen University, Guangzhou, 501180 China; 2 Key Laboratory of Tropical Disease Control of Ministry of Education, Sun Yat-Sen University, Guangzhou, 501180 China; 3 Guangdong Engineering Research Center for Antimicrobial Agent and Immunotechnology, Sun Yat-Sen University, Guangzhou, 510080 China; 4 Department of Infectious Diseases, Nanfang Hospital, Southern Medical University, Guangzhou, 510515 China; 5 Department of Infectious Diseases, The Fifth Affiliated Hospital of Sun Yat-sen University, Zhuhai, 519000 China; 6 Program in Pathobiology, The Fifth Affiliated Hospital and Zhongshan School of Medicine, Sun Yat-sen University, Zhuhai, 519000 China

## Abstract

Hepatitis C virus (HCV) infection is a major cause of chronic hepatitis and liver cancer worldwide. Adaptive mutations play important roles in the development of the HCV replicon and its infectious clones. We and others have previously identified the p7 mutation F772S and the co-presence of NS4A mutations in infectious HCV full-length clones and chimeric recombinants. However, the underlying mechanism of F772S function remains incompletely understood. Here, we investigated the functional role of F772S using an efficient JFH1-based reporter virus with Core-NS2 from genotype 2a strain J6, and we designated J6-p7/JFH1-4A according to the strain origin of the p7 and NS4A sequences. We found that replacing JFH1-4A with J6-4A (wild-type or mutated NS4A) or genotype 2b J8-4A severely attenuated the viability of J6-p7/JFH1-4A. However, passage-recovered viruses that contained J6-p7 all acquired F772S. Introduction of F772S efficiently rescued the viral spread and infectivity titers of J6-p7/J6-4A, which reached the levels of the original J6-p7/JFH1-4A and led to a concomitant increase in RNA replication, assembly and release of viruses with J6-specific p7 and NS4A. These data suggest that an isolate-specific cooperation existed between p7 and NS4A. NS4A exchange- or substitution-mediated viral attenuation was attributed to the RNA sequence, and no p7-NS4A protein interaction was detected. Moreover, we found that F772S-enhanced p7-NS4A cooperation was associated with the enlargement of intracellular lipid droplets. This study therefore provides new insights into the mechanisms of adaptive mutations and facilitates studies on the HCV life cycle and virus–host interaction.

## Introduction

Hepatitis C virus (HCV) chronically infects 71 million people worldwide according to the estimation of World Health Organization^1^. HCV infection can lead to chronic hepatitis C, which increases the risk of developing liver fibrosis, cirrhosis, and hepatocellular carcinoma^2,3^. To date, no HCV vaccine is available. Recently, the use of direct-acting antiviral agents (DAAs) has revolutionized HCV therapy and cured ≥ 90% of patients^4^. However, pegylated interferon-α in combination with ribavirin (Peg-IFN/RBV) is still the standard of care for hepatitis C in many countries and/or regions^5^, which has unfavorable adverse effects and only cures ~50% of patients^6^. Thus, challenges for hepatitis C treatment remain regarding the introduction of more-effective regimens, especially in DAA-based therapy, more patients who are in need and in optimizing regimens, limiting drug resistance, and ultimately providing a preventative vaccine.

HCV belongs to the *hepacivirus* genus of the Flaviviridae family. The HCV genome is a positive-sense, single-stranded RNA genome of ~9600 nucleotides that consists of a 5′-untranslated region (UTR), an open reading frame (ORF), and a 3′UTR. The ORF is translated into a polyprotein of 3000 amino acids (aa), which is processed into 10 viral proteins by host peptidase and viral proteases, including three structural proteins (Core, E1, and E2), p7, and six nonstructural proteins (NS2, NS3, NS4A, NS4B, NS5A, and NS5B)^7,8^. The structural proteins constitute the viral particle, and the nonstructural proteins are critical for virus replication and other steps of the viral life cycle.

HCV p7 is a small viroporin of 63 amino acids and spans the endoplasmic reticulum (ER) membrane by two transmembrane domains connected by a cytoplasmic loop^9^. The p7 protein is not required for HCV RNA replication^10^, but is required for the entry, assembly, release, envelopment, and production of infectious virions;^11–13^ it is also critical for cell-to-cell transmission of viral RNA or genomic material^14^. Nevertheless, the function of p7 in the HCV life cycle is not fully understood.

NS4A is a single transmembrane protein of 54 amino acids. The N-terminal region (aa 1–20) of NS4A is required for association with the ER membrane^15^, whereas the central part of NS4A is more variable in sequence, containing the NS3 cofactor (aa 21–32) and a kink region (aa 33–39). The C-terminal domain (aa 40–54) is conserved and has been shown to modulate HCV RNA replication^16^. As a part of the NS3/4 A protease, NS4A may be involved in the regulation of the innate immune response through cleavage of the antiviral mitochondrial signaling adaptor MAVS^17–19^.

The development of replicon and infectious cell culture systems has greatly accelerated HCV research^10,20–22^. A number of adaptive mutations have been identified important for HCV replication and virus production^23–31^. Previously, we identified LSG mutations [F1464L in NS3, A1672S (NS4A), and D2979G (NS5B)]^24^. The amino-acid positions in this manuscript are given according to the H77 reference strain (GenBank number AF009606)^32^. The LSG enabled the development of full-length infectious HCV clones of genotypes 2a (J6cc), 2b (J8cc, DH8cc, and DH10cc), and 1a (TNcc, H77Ccc, and HCV1cc)^24,26,27,29^ as well as 5′UTR-NS5A recombinants of genotypes 3a, 4a, 5a, and 6a^25^. Notably, the mutation F772S in the transmembrane domain of p7 protein was frequently identified in full-length HCV infectious clones^23,24^ and replicon systems^33^. However, the functional role of the adaptive mutation F772S remains unclear.

Here, we demonstrated that p7 cooperated with NS4A in an isolate-specific manner. F772S enhanced the cooperation and facilitated viral spread and assembly/release, which were associated with the enlargement of cellular lipid droplets (LD).

## Results

### Mutation F772S compensated for the viral attenuation that resulted from NS4A replacement, implying a p7-NS4A cooperation

**Fig. 1:**
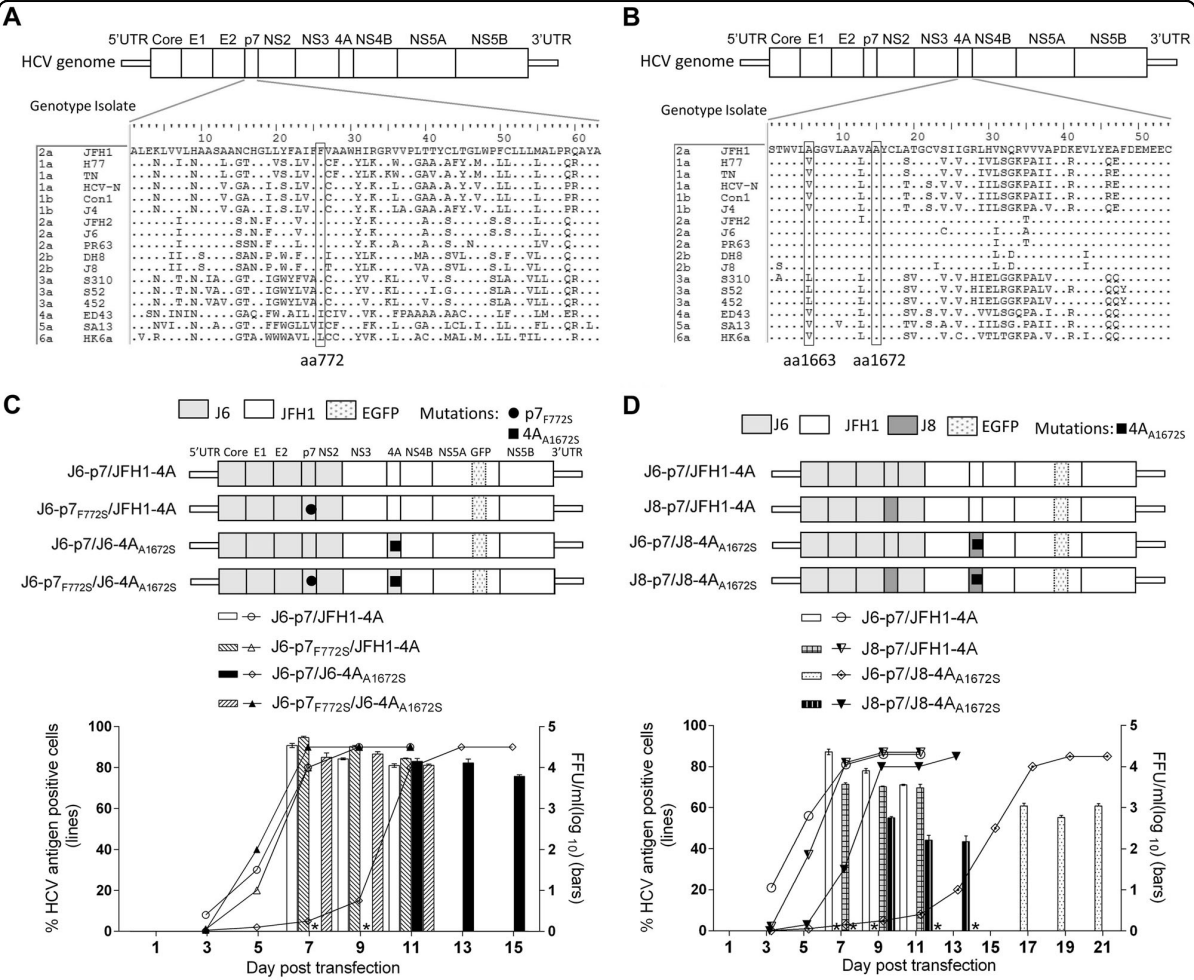
Isolate-specific p7 and F772S mutation rescued the viral attenuation caused by NS4A substitutions. **a**, **b** Alignments of p7 and NS4A proteins of HCV genotypes 1–6. **c**, **d** Transfection of HCV recombinants with different mutations. The HCV antigen was detected by immunostaining or EGFP, and the percentage of virus positive cells was estimated (left *y* axis). HCV supernatant infectivity titers at peak infection were determined by the FFU assay (right *y* axis), and mean ± SEM of three determinations is shown. *, FFU was not determined owing to its low infection rate

Previously, we found that F772S (p7) was required for efficient production of LSG-based genotype 2a J6cc clone^24^. The co-presence of F772S and A1672S (NS4A, the “S” of LSG) allowed the replication of J6-JFH1 chimera J6(JFH1_5BthuX)^24^. In addition, J6/JFH1 containing NS4A of genotypes 1a, 1b, 4a, 5a, 6a, and 7a all acquired F772S and V1663A (NS4A) for efficient production^23^. Thus, we hypothesized that p7 may cooperate with NS4A to enhance virus production. To address this question, we selected an efficient intragenotypic 2a reporter virus, J6/JFH1-EGFPΔ40, as a constant backbone^34^. As J6/JFH1-EGFPΔ40 contains J6 Core-NS2 and JFH1 5′UTR/NS3-3′UTR, we designated it as J6-p7/JFH1-4A for simplicity. The alignment of p7 and NS4A proteins of genotypes 1–6 are shown in Fig. 1a, b.

To study whether F772S increases virus production of J6-p7/JFH1-4A, we constructed J6-p7_F772S_/JFH1-4A (Fig. 1c). After RNA transfection into Huh7.5 cells, the percentage of HCV Core or NS5A-EGFP-positive cells was determined. Both J6-p7/JFH1-4A and J6-p7_F772S_/JFH1-4A viruses spread to ≥ 80% of cultured cells (peak infection) at day 7 and released supernatant HCV infectivity titers of 10^4.3^–10^4.7^ focus forming units (FFU)/ml. The culture supernatants at peak infection were collected and inoculated to naive Huh7.5 cells. After the virus reached peak infection, the culture supernatant was collected (first-passage virus). Sequence analysis of the p7 and NS4A of first-passage viruses revealed no additional changes, whereas F772S was maintained in J6-p7_F772S_/JFH1-4A (Table 1). These results suggest that the F772S did not affect the virus spread and infectivity titers of J6-p7/JFH1-4A, indicating that the wild-type and F772S-contianing J6-p7 had a similar capacity for the replication of recombinants containing JFH1-4A.

**Table 1:** Sequence analysis of first-passage HCV recombinants with different p7 and NS4A sequences, with or without engineered mutations

HCV gene	Peak infectivity titers in transfections (FFU/ml, log_10_)(Day)	First-passage sequenced (Day)	p7	NS4A	NS4A
Nucleotide position					
Recombinant specific			2667	5340	5366
H77 reference (AF009606)			2656	5329	5355
Recombinant nucleotide			T	C	A
J6-p7/JFH1-4A, exp. 1^*^	4.5 (7)	9	T/c	.	.
J6-p7 /JFH1-4A, exp. 2	4.3 (7)	9	T/c	.	.
J6-p7_F772S_/JFH1-4A, exp. 1^*^	4.7 (7)	7	C	.	.
J6-p7_F772S_/JFH1-4A, exp. 2	4.1 (9)	9	C	.	.
J6-p7/J6-4A_A1672S_, exp. 1*	4.1 (11)	7	C/t	.	T
J6-p7/J6-4A_A1672S_, exp. 2	3.5 (13)	9	C/t	.	T
J6-p7_F772S_/J6-4A_A1672S_, exp. 1^*^	4.4 (7)	7	C	.	T
J6-p7_F772S_/J6-4A_A1672S_, exp. 2	3.4 (9)	9	C	.	T
J8-p7/JFH1-4A^#^	3.5 (9)	7	.	.	.
J6-p7/J6-4A	3.8 (9)	9	T/c	.	.
J6-p7/J8-4A_A1672S_	3.0 (17)	7	T/c	.	T
J8-p7/J8-4A_A1672S_^#^	2.7 (9)	7	.	.	T
J6-p7/J6-4A_A1663V/A1672S_	3.6 (13)	9	T/c	T	T
J6-p7_F772S_/J6-4A_A1663V/A1672S_	3.5 (11)	9	C	T	T
J6-p7_F772S_/J6-4A	3.7 (7)	9	C	.	.
Amino-acid position					
Recombinant specific			776	1667	1676
H77 reference (AF009606)			772	1663	1672
Change			F-S	A-V	A-S

The nucleotide and amino-acid positions of the specific recombinant with mutations are given, with corresponding position of H77 sequence (AF009606). Shade, engineered mutations. Dominant/minor (capital/lowercase) nucleotides in sequencing reads are shown

“.” indicates identical to the original nucleotide, ^*^similar results were observed in experiment 2. ^#^, the p7 in J8 contained F768C substitution, important for the viability of the virus

Next, we replaced the JFH1-4A of J6-p7/JFH1-4A or J6-p7_F772S_/JFH1-4A into J6-4A_A1672S_. Of note, A1672S is essential for the function of J6-NS4A in the J6cc^24^. The resultant J6-p7/J6-4A_A1672S_ was severely attenuated and spread to peak infection at day 11 (peak titer of 10^4.1^ FFU/ml), whereas J6-p7_F772S_/J6-4A_A1672S_ reached peak infection at day 7 (10^4.4^ FFU/ml), which were comparable to the original J6-p7/JFH1-4A (10^4.6^ FFU/ml) (Fig. 1c). First-passage J6-p7/JFH1-4A and J6-p7/J6-4A_A1672S_ did not acquire additional changes in NS4A; however, F772S was emerged in both viruses and became dominant in J6-p7/J6-4A_A1672S_. J6-p7_F772S_/J6-4A_A1672S_ had no changes (Table 1). These data demonstrate that F772S in p7 rescued the viral attenuation caused by substitutions of NS4A, implying a cooperation between p7 and NS4A.

### p7 cooperated with NS4A in an isolate-specific manner to enhance virus production

We also constructed recombinants with p7, NS4A, or both p7 and NS4A from the genotype 2b clone J8cc to make J8-p7/JFH1-4A, J6-p7/J8-4A_A1672S_, and J8-p7/J8-4A_A1672S_, respectively (Fig. 1d). A1672S is essential for the function of the J8 NS4A sequence^29^, thus it was kept in the recombinant. In RNA transfection of Huh7.5 cells, J8-p7/JFH1-4A was delayed in viral spread (20% vs 2% at day 3) and detectable infectivity titers (day 9 vs day 7) compared with J6-p7/JFH1-4A (Fig. 1d); the peak infectivity titers were also lower by ~1 log_10_ FFU/ml. Thus, J8-p7 had a lower efficiency than J6-p7 in terms of virus production. J6-p7/J8-4A_A1672S_ showed only 20% HCV-positive cells at day 13 (infectivity titer undetectable), whereas in contrast J8-p7/J8-4A_A1672S_ was 80% at day 9 (10^2.7^ FFU/ml) (Fig. 1d). First-passage viruses did not acquire additional changes in p7 and NS4A (Table 1). These results indicate that the increased viability of J8-p7/J8-4A_A1672S_ was owing to the co-presence of J8-p7 and J8-4A_A1672S_, and thus, p7 cooperated with NS4A in an isolate-specific manner to enhance virus production.

### F772S compensated for the attenuation resulting from substitutions of A1663V and A1672S in NS4A

**Fig. 2:**
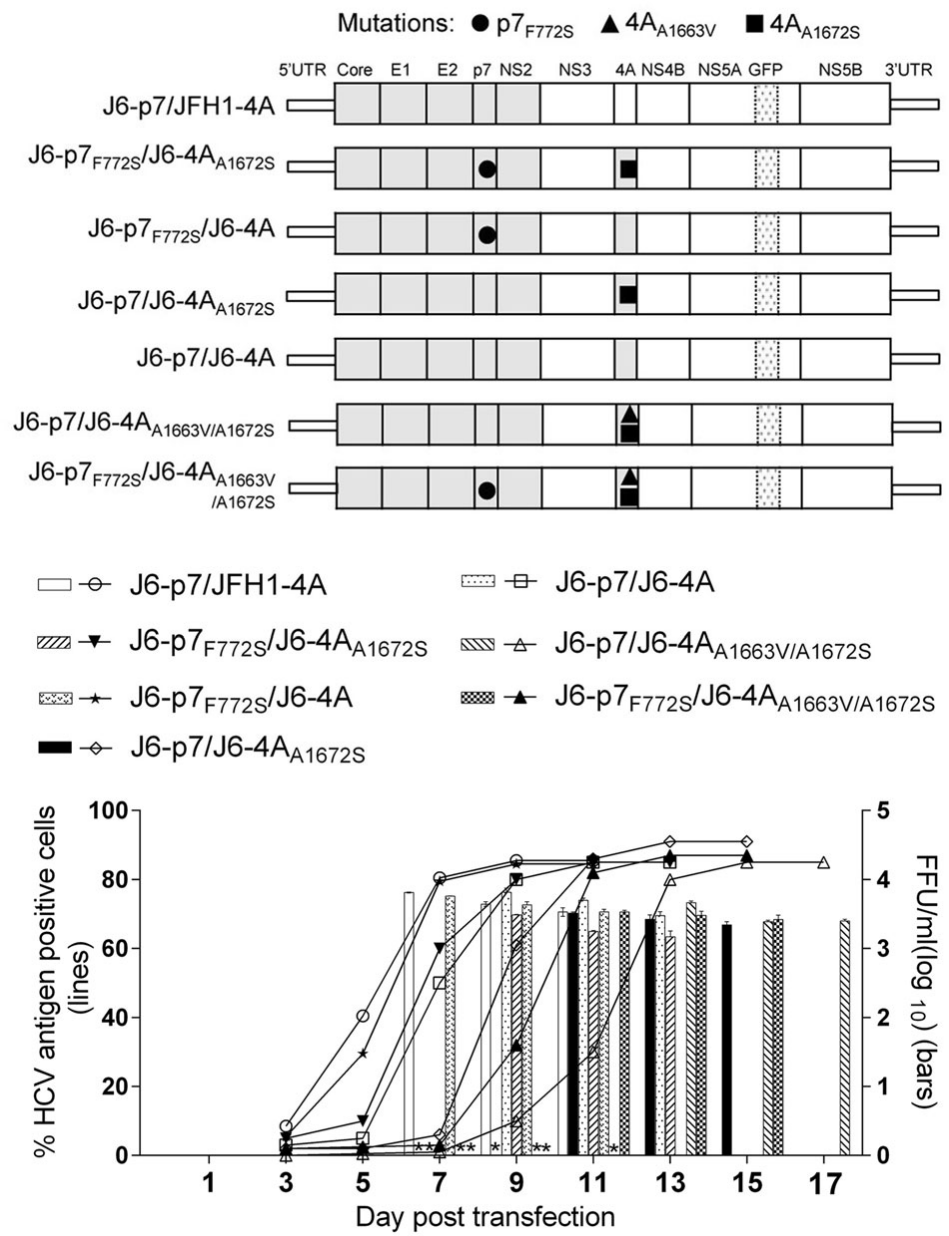
F772S compensated for the attenuation caused by A1663V and A1672S substitutions in NS4A. Transfection of HCV recombinants with different mutations. The percentage of infected cells and HCV supernatant infectivity titers were shown. See Fig. 1 legend for details

As replacing JFH1-4A into J6-4A_A1672S_ led to viral attenuation (Fig. 1c), we further studied whether the attenuation was due to A1672S. We mutated S1672 back to wild-type A1672 to generate J6-p7/J6-4A and J6-p7_F772S_/J6-4A (Fig. 2). In equal amount of RNA transfections, J6-p7_F772S_/J6-4A spread more efficiently than J6-p7_F772S_/J6-4A_A1672S_, reaching its peak infection and detectable infectivity titer at days 7 and 9, respectively. Likewise, J6-p7/J6-4A spread faster than J6-p7/J6-4A_A1672S_, reaching its peak infection and measurable infectivity titers at days 9 and 11, respectively. These results revealed that A1672S was unfavorable for the viability of the virus; however, the addition of F772S enhanced viral spread. Thus, F772S rescued the viral attenuation caused by A1672S, suggesting that A1672 was important for the viability of the recombinant viruses.

Previously, the combination of F772S and V1663A was found to be important for the adaptation of J6/JFH1 with NS4A of genotypes 1a, 1b, 4a, 5a, and 6a, in which V1663 is conserved^23^. A1663 is conserved in genotype 2a and 2b (Fig. 1b). To study whether F772S cooperates with A1663, we introduced A1663V into J6-p7/J6-4A_A1672S_ and found that J6-p7/J6-4A_A1663V/A1672S_ was slower in virus spread than J6-p7/J6-4A_A1672S_, reaching peak infection and detectable infectivity titers on days 13 and 11, respectively. However, the introduction of F772S (J6-p7_F772S_/J6-4A_A1663V/A1672S_) increased the kinetics of virus spread, showing the enhancement effect of F772S. Hence, A1663V was also an unfavorable change for J6-p7/J6-4A_A1663V/A1672S_, and F772S was able to compensate for the adverse effects of A1663V, suggesting that A1663 was important for the viability of the recombinant viruses.

### F772S coordinated with NS4A by enhancing viral RNA replication

**Fig. 3:**
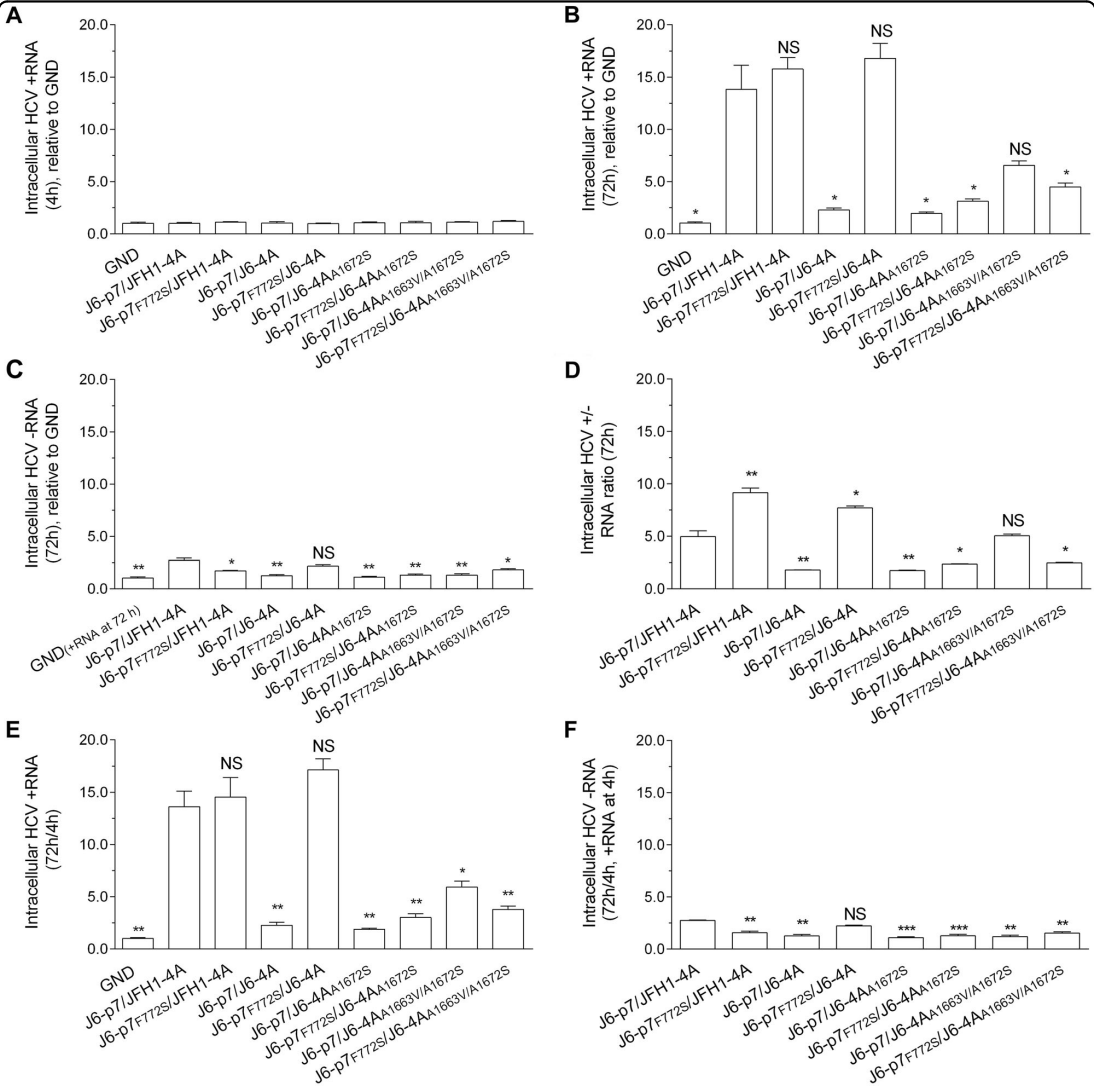
F772S-enhanced RNA replication of recombinants with isolate-specific p7 and NS4A. RNA transcripts (10 μg) of different HCV recombinants were transfected into Huh7.5 cells, and the cells were collected at 4 and 72 h p.t. J6/JFH1-EGFP△40-GND (GND) was the control. **a** HCV +RNA at 4 h p.t. was determined (baseline). **b**, **c** Fold-change of HCV +RNA and -RNA at 72 h relative to GND. **d** The ratio of +/-RNA at 72 h. **e**, **f** Fold-change of + RNA and -RNA at 72 h (normalized to 4 h +RNA). The data presented in (**a–f**) are the average ± SEM of three determinations, and three independent experiments were performed. Statistical significance was calculated against J6-p7/JFH1-4A and is indicated by asterisk(s) (*, *p* ≤ 0.05; **, *p* ≤ 0.01; ***, *p* ≤ 0.001). NS, no significant difference (student’s *t* test)

To explore the mechanisms underlying the compensatory effect of F772S, we performed a short-term transfection assay (72 h) to minimize and ignore the impact of mutations possibly emerged. We quantified the relative amount of intracellular HCV-positive RNA (+RNA) and negative RNA (−RNA; an indicator of active HCV replication) in three independent experiments and obtained similar results (Fig. 3). The +RNA levels at 4 h post transfection (p.t.) were set as the input baseline RNA and were close to the replication-deficient J6-p7/JFH1-4A_GND control (Fig. 3a). The +RNA and –RNA levels of recombinants at 72 h was relative to the GND +RNA level (no –RNA for GND) (Fig. 3b, c). At 72 h p.t., the +RNA levels of different recombinants were increased by 2.5–17-fold compared with GND, whereas the −RNA levels were increased by ~2.5-fold or less (Fig. 3b, c). The ratios of ±RNAs were 2–9-fold, of which the ratio for J6-p7_F772S_/J6-4A and J6-p7_F772S_/JFH1-4A were similar, and both were higher than other recombinants (Fig. 3d). Addition of F772S significantly increased the +RNA level of J6-p7/J6-4A, as the +RNA level of J6-p7_F772S_/J6-4A was approximately seven-fold higher than that of J6-p7/J6-4A (Fig. 3b).

To further examine the increase in either +RNA or −RNA, we normalized the +RNA and –RNA at 72 h to +RNA at 4 h (72 h/4 h) (4 h −RNA undetectable) (Fig. 3e, f). Overall, the increased +RNA and −RNA levels were similar to those when it was normalized to GND (Fig. 3b–f). The 72 h/4 h +RNA varied from 2.0–17.1-fold; J6-p7/JFH1-4A (13.6 fold) and J6-p7_F772S_/JFH1-4A (14.5-fold) were similar, whereas J6-p7/J6-4A (2.3-fold) was significantly lower than J6-p7_F772S_/J6-4A (17.1-fold). Other recombinants were more than sixfold (Fig. 3e). The 72 h/4 h −RNA was all < 2.5-fold (Fig. 3f).

Taken together, these data suggest that F772S promoted the replication of the viruses with isolate-specific NS4A. However, the enhancement effect of F772S was not observed or only minor at 72 h when NS4A contained A1672S or A1663V/A1672S.

### F772S enhanced the replication, assembly/release of HCV recombinants with J6-specific p7 and NS4A

**Fig. 4:**
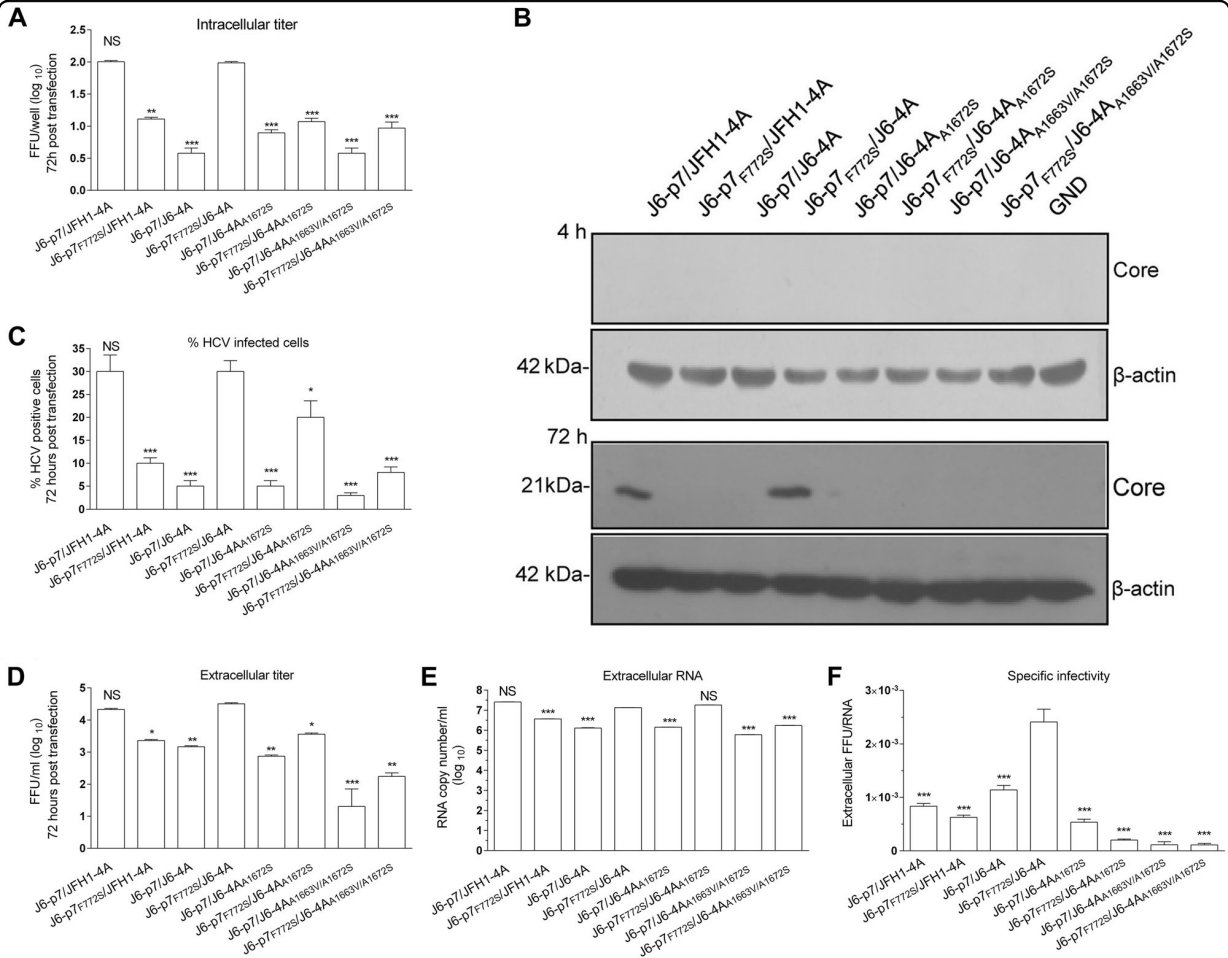
F772S enhanced the assembly/release of HCV recombinants with isolate-specific p7 and NS4A. Huh7.5 cells were transfected with RNA transcripts (10 μg) of different HCV recombinants, and the analyses were performed at 72 h p.t. **a** Intracellular titers were determined (FFU/well). **b** Western blotting of intracellular HCV Core. **c** The HCV infection rate was estimated by core immunostaining or NS5A-EGFP expression. **d** Extracellular infectivity titers (FFU/ml). **e** Extracellular RNA titers (copy/ml). **f** Specific infectivity (FFU/RNA ratio). See Fig. 3 legend for the detailed annotations

It is known that p7 is crucial for the assembly and release of infectious HCV particles^11–13^. To investigate whether F772S plays a role in these steps, we performed short-term transfection experiment. After 72 h of RNA (10 μg) transfections, we analyzed the infection rate, intracellular infectivity titers, core protein, extracellular infectivity, RNA titers, and specific infectivity (Fig. 4).

The results of the intracellular titers showed that F772S decreased the assembly of infectious particles of the J6-p7/JFH1-4A, as J6-p7_F772S_/JFH1-4A was ~1 log_10_ FFU/ml lower than J6-p7/JFH1-4A (Fig. 4a). Replacement of JFH1-4A with J6-4A or J6-4A_A1672S_ decreased the formation of infectious particles; however, addition of F772S increased the intracellular titer of J6-p7/J6-4A, J6-p7/J6-4A_A1672S_, and J6-p7/J6-4A_A1663V/A1672S_. J6-p7_F772S_/J6-4A was comparable to J6-p7/JFH1-4A, and both were the most efficient viruses (Fig. 4a). These results were further confirmed by western blot, in which HCV Core was detectable only for J6-p7/JFH1-4A and J6-p7_F772S_/J6-4A (Fig. 4b).

The infection rate and infectivity titers of J6-p7_F772S_/J6-4A were also comparable to that of J6-p7/JFH1-4A (30% HCV-infected cells, 10^4.3^ FFU/ml) (Fig. 4c, d). However, J6-p7_F772S_/JFH1-4A (10%, 10^3.2^ FFU/ml), J6-p7/J6-4A (5%, 10^3.4^ FFU/ml), and J6-p7/J6-4A_A1672S_ (5%, 10^2.9^ FFU/ml) showed less HCV-positive cells and infectivity titers, suggesting that introduction of F772S or replacement of J6-4A or J6-4A_A1672S_ impaired J6-p7/JFH1-4A. A1663V change further impaired J6-p7/J6-4A_A1672S_ as J6-p7/J6-4A_A1663V/A1672S_ showed only 2–3% HCV-positive cells and 10^1.3^ FFU/ml. By contrast, addition of F772S to those recombinants with J6-4A or J6-4A_A1672S_ increased the infection rate, with 30% HCV-positive cells for J6-p7_F772S_/J6-4A (10^4.5^ FFU/ml) and 20% for J6-p7_F772S_/J6-4A_A1672S_ (10^3.5^ FFU/ml). The infectivity titer of J6-p7_F772S_/J6-4A was nearly 30-fold higher than that of J6-p7/J6-4A_A1663V/A1672S_, 21-fold higher than that of J6-p7/J6-4A, and six-fold higher than that of J6-p7/J6-4A_A1672S_. Thus, F772S increased the virus spread and production. These results confirmed the observations in the transfection experiments with long-term follow-up, in which F772S rescued the attenuation resulted from J6-4A or point mutations (Figs. 1 and 2). It should also be noted that the co-presence of S772 and A1663 or A1672 was important for virus production (Fig. 2). Since J6-p7_F772S_/J6-4A was more efficient than J6-p7_F772S_/JFH1-4A at 72 h p.t. (infection rate, 30 vs 10%; FFU/ml, 10^4.3^ vs 10^3.3^), these results from the early time point further confirmed that F772S enhanced the viability of recombinants with isolate-specific p7 and NS4A.

The extracellular RNA level of J6-p7_F772S_/J6-4A was 1.3 × 10^7.0^ copies/ml, which was similar to that of J6-p7/JFH1-4A and J6-p7_F772S_/J6-4A_A1672S_ but was 10-fold higher than that of other recombinants (Fig. 4e). The specific infectivity (ratio of FFU/+RNA) of a virus is an indicator of the quality of virus assembly; thus, we also calculated the specific infectivity of supernatant viruses (Fig. 4f). The specific infectivity of J6-p7_F772S_/J6-4A was 20-fold higher than that of J6-p7/J6-4A_A1663V/A1672S_ and J6-p7_F772S_/J6-4A_A1663V/A1672S_ and 2.5-fold higher than that of J6-p7/J6-4A (Fig. 4f), suggesting that the introduction of F772S enhanced the assembly of J6-p7/J6-4A.

Taken together, the results from short-term assays demonstrated that F772S enhanced the assembly and release of HCV recombinants containing isolate-specific p7 and NS4A.

### F772S-enhanced p7-NS4A cooperation was at the NS4A RNA sequence level and not at the protein level

Next, we proceeded to investigate whether p7 physically interacts with NS4A at the protein level. We overexpressed HA- or Flag-tagged J6-p7 or J6-NS4A (with or without mutations). However, we did not observe a direction interaction of J6-p7 and J6-NS4A in immunoprecipitation, even after several experimental optimizations. Thus, we assumed that other viral proteins or host factors may be required to mediate p7-NS4A interaction in Huh7.5 cells during the complete HCV life cycle.

**Fig. 5:**
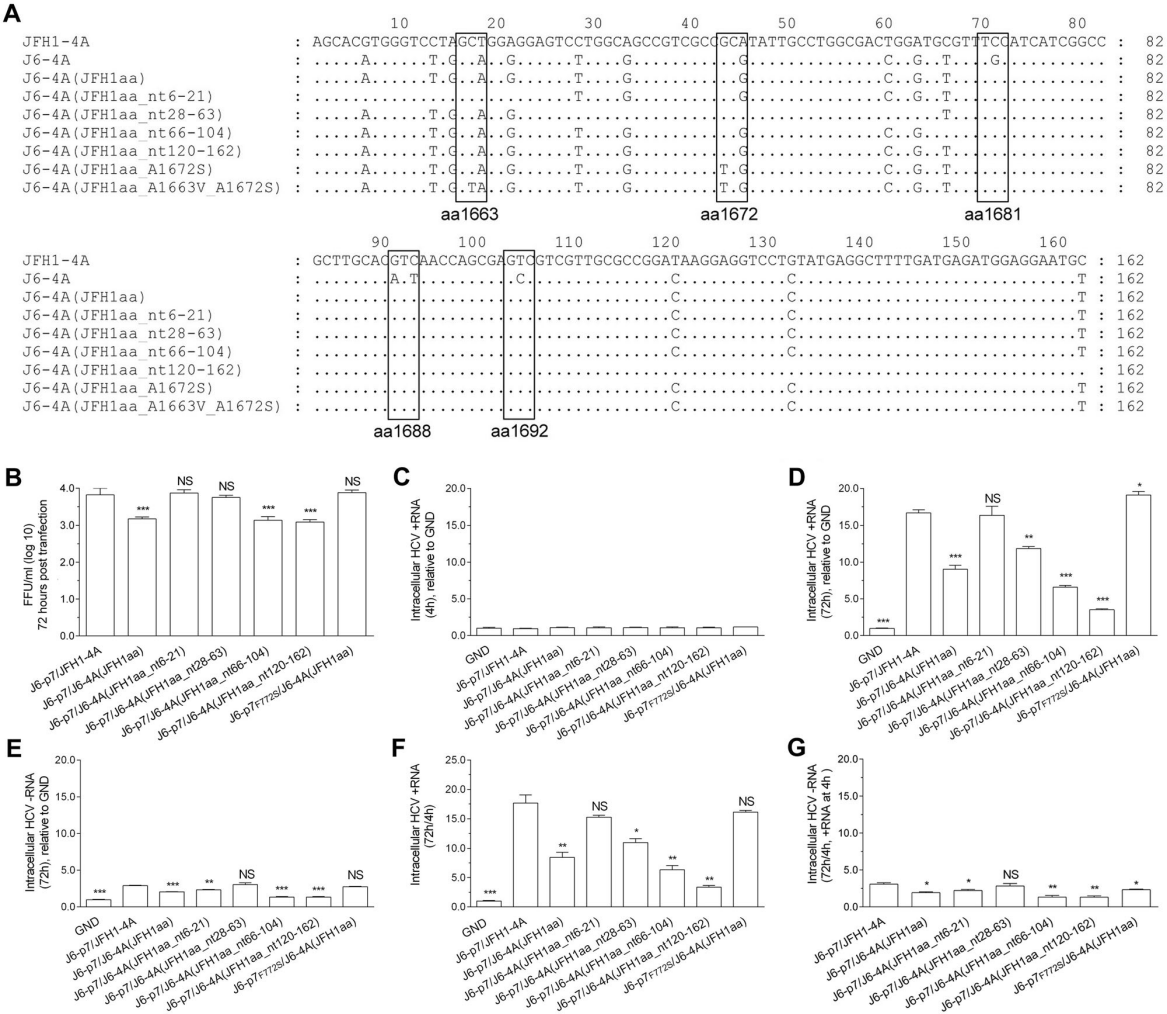
F772S-enhanced p7-NS4A cooperation involved the NS4A RNA sequence. Recombinants expressing JFH1-type NS4A were transfected into Huh7.5 cells, and the analyses were performed at 4 h and 72 h p.t. **a** RNA sequence of J6-JFH1 NS4A chimeras. **b** The infectivity titers of JFH1 NS4A-expressing recombinants. **c** HCV +RNA at 4 h p.t. (baseline). **d**, **e** Fold-change of +RNA and -RNA at 72 h p.t. relative to GND. **f**, **g** Fold-change of +RNA and -RNA at 72 h (normalized to 4 h +RNA). The data in **c–g** are the average ± SEM (*n* = 3). See Fig. 3 legend for the detailed annotations

As replacing JFH1 NS4A into J6-NS4A attenuated the virus (Figs. 1 and 2), we further studied whether this attenuation was owing to differences in the NS4A protein. Only 18 nucleotides and three amino-acid differences exist in NS4A sequence between JFH1 and J6 isolates (Fig. 5a). We introduced C1681S, I1688V, and A1692V into J6-p7/J6-4A to constitute a JFH1-type NS4A protein, but with a different RNA sequence, and we designated this recombinant J6-p7/J6-4A(JFH1aa). Meanwhile, based on J6-p7/J6-4A(JFH1aa), we divided the 18 different nucleotides into four fragments (nt6-21, 28–63, 66–104, and 120–162) and mutated each fragment to JFH1 nucleotides (Fig. 5a). As shown in Fig. 5b, J6-p7/J6-4A(JFH1aa) was ~0.5 log_10_ FFU/ml lower than J6-p7/JFH1-4A. Substitutions of nt6-21 and nt28-63 into JFH1 nucleotides increased the infectivity titers of J6-p7/J6-4A(JFH1aa) to the level of J6-p7/JFH1-4A, while the nt66-104 and nt120-162 viruses were similar to J6-p7/J6-4A(JFH1aa). Introduction of F772S into J6-p7/J6-4A(JFH1aa) increased its infectivity to the level of J6-p7/JFH1-4A. These results suggest that J6-4A-mediated attenuation involved RNA sequence, and nucleotides 6–63 accounted for the attenuation.

To determine whether the JFH1aa-coding NS4A RNA sequences affect the viral genome replication, we quantified the intracellular viral +RNA and −RNA levels. At 4 h p.t., the input RNA levels were similar among the recombinants (Fig. 5c). At 72 h, the +RNA levels of J6-p7/J6-4A(JFH1aa), J6-p7/J6-4A(JFH1aa_nt66-104),andJ6-p7/J6-4A(JFH1aa_nt120-162) were increased up to ~3–9-fold but lower than that of J6-p7/JFH1-4A (~17-fold) and J6-p7/J6-4A(JFH1aa_nt28-63) (~12.5-fold), whereas J6-p7/J6-4A(JFH1aa_nt6-21) was comparable to original J6-p7/JFH1-4A (Fig. 5d). Addition of F772S increased the viral RNA level of J6-p7/J6-4A(JFH1aa) up to the level of J6-p7/JFH1-4A. The −RNA levels were increased more than threefold (Fig. 5e). Moreover, we also examined the increase of +RNA and –RNA by normalizing to the respective +RNA level at 4 h (Fig. 5f, g). The fold changes of +RNA and –RNA were similar to the results that RNA levels were related to GND (Fig. 5d, e). Together, nucleotides 6–63 of J6-4A involved in F772S-enhanced p7-NS4A cooperation were crucial for viral RNA replication.

### F772S-enhanced viral assembly/release was associated with an enlargement of LDs in HCV-infected cells

**Fig. 6:**
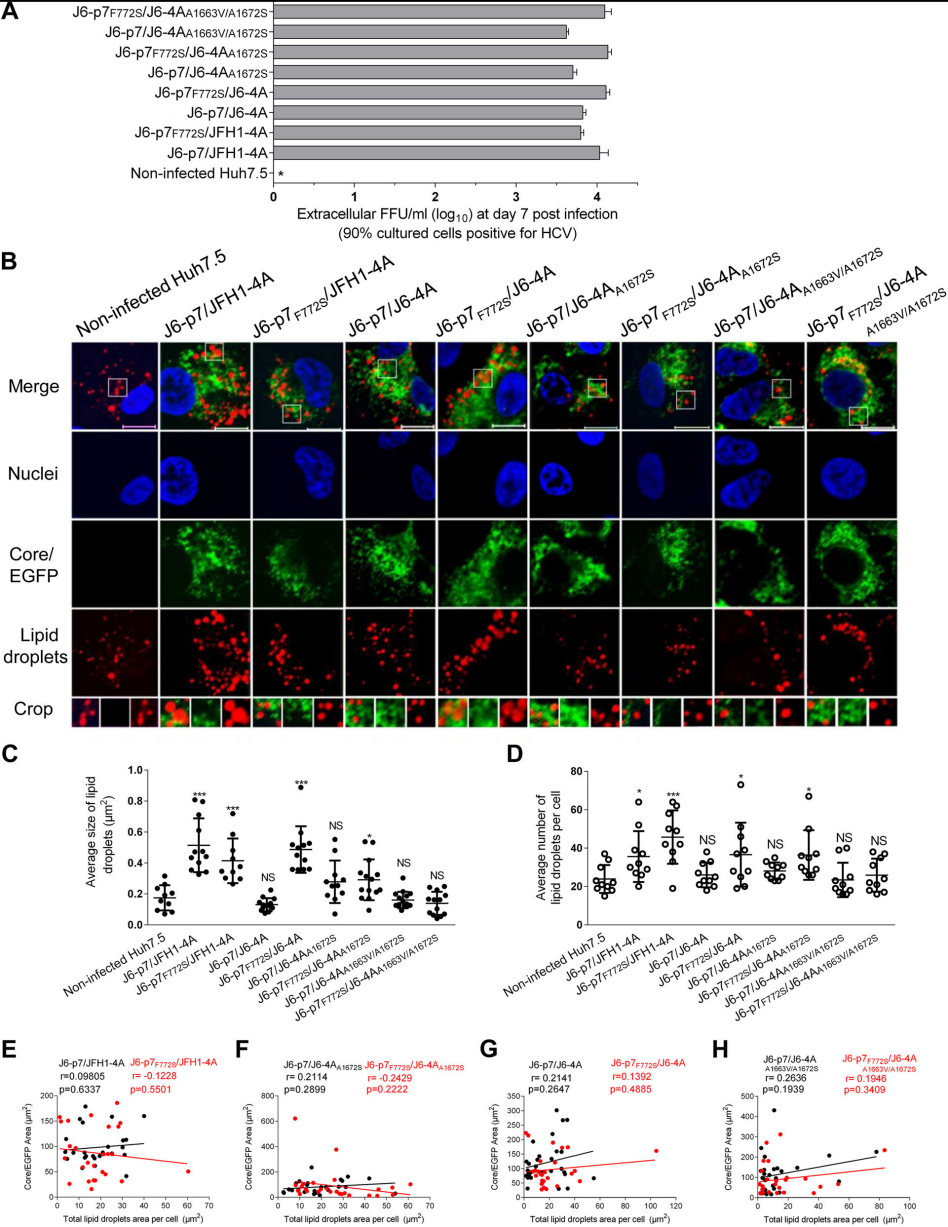
F772S-enhanced viral assembly/release was associated with the enlargement of LDs in HCV-infected cells. Huh7.5 cells were infected with HCV (MOI = 0.1), and ~90% of cells were HCV positive at day 7. The cells were fixed and stained for HCV and LDs. **a** The supernatant infectivity titers at day 7 post infection. **b** Images of HCV antigens (Core/NS5A-EGFP) (green), nuclei (blue), LD (red), and the merged images. Bar, 10 μm. **c** The average size of LDs in HCV positive Huh7.5 cells (10–15 cells). **d** The average numbers of LDs per Huh7.5 cell positive for HCV (by ImageJ software). See Fig. 3 legend for the annotations of statistical analysis. **e**–**h** The correlation analysis of the total HCV protein and the formation of LDs in 26 HCV-infected cells using ImageJ software. Correlation coefficient (*r* value) and *p* value are shown. *p* ≤ 0.05 indicates a statistically correlation, in which the higher *r* value suggests the stronger correlation

It is known that LDs are important organelles associated with HCV assembly and production of infectious virus particles^35^. AS p7-NS4A cooperation enhanced viral assembly, we set out to determine whether this effect is associated with the LDs. We performed Oil Red O staining coupled with immunostaining procedures to analyze the size and number of LDs following HCV infection. To minimize the bias of the infection rate on the lipid production, we analyzed the lipid content when the culture reached peak infection (≥90% cells positive for HCV at day 7 post infection) (Fig. 6). The results showed that infectivity titers of HCV recombinants were similar (<0.5 log_10_ FFU/ml) (Fig. 6a). We randomly determined the size of the LDs in 10–15 cells and found that the LDs in non-infected control and J6-p7/JFH1-4A-infected Huh7.5 cells were 0.17 μm^2^ and 0.51 μm^2^, respectively. Replacement of J6-4A severely reduced the size of LDs (0.13 μm^2^) (Fig. 6a, b). The size of LDs for J6-p7_F772S_/J6-4A infection (0.49 μm^2^) was comparable to that infected with J6-p7/JFH1-4A (0.51 μm^2^). Addition of F772S partially restored the viability of the viruses with isolate-specific p7 and NS4A; however, F772S did not significantly alter the LDs of J6-p7/JFH1-4A (J6-p7/JFH1-4A vs J6-p7_F772S_/JFH1-4A). Substitution of A1672S and A1663V/A1672S reduced the size of LDs (0.28 μm^2^ and 0.14 μm^2^, respectively), and this size reduction could not be rescued by addition of F772S, indicating that the co-presence of S772/A1672 or S772/A1663/A1672 was important for the growth of the LDs (Fig. 6b).

The numbers of LDs following the infections of J6-p7/J6-4A, J6-p7/J6-4A_A1672S_, J6-p7/J6-4A_A1663V/A1672S_, and J6-p7_F772S_/J6-4A_A1663V/A1672S_ were not significantly different from the non-infected Huh7.5 cells. J6-p7_F772S_/J6-4A and J6-p7_F772S_/JFH1-4A induced more LDs, as did J6-p7/JFH1-4A (Fig. 6c). Thus, these data suggest that p7-NS4A cooperation-enhanced viral assembly was associated with lipid growth and formation, in which S772, A1663, and A1672 played a role.

To clarify whether the difference in the enlargement of LDs was the consequence of difference in HCV protein levels, we quantitatively analyzed the viral proteins in the infected cells, in which the size and numbers of LDs were determined. No correlation was identified between the amount of HCV protein and the size and number of LDs (data not shown). These observations were further confirmed by randomly analyzing 26 HCV-positive cells. Indeed, no correlation was found (Fig. 6e–h). For example, J6-p7/J6-4A and J6-p7_F772S_/J6-4A associated with a significant difference in the size and numbers of LDs, the correlation coefficient (*r* value) was 0.1392 (*p* = 0.4885), thus suggesting a non-correlation between HCV protein levels and the changes in LDs (Fig. 6g). In addition, taking into account no difference in the LDs between HCV-infected (e.g., J6-p7/J6-4A and other NS4A-mutated viruses) and non-infected cells (Fig. 6c, d), the presence of HCV protein or difference in HCV protein levels did not affect the formation of LDs. Therefore, the changes in the LDs were most likely the consequence of the F772S enhanced p7-NS4A cooperation.

## Discussion

Adaptive mutations significantly contribute to the development of infectious culture systems that recapitulate the complete life cycle of HCV. In this study, we demonstrated that p7 cooperated with NS4A in an isolate-specific manner. F772S enhanced the cooperation and facilitated viral spread and assembly/release, which are associated with the enlargement of cellular LDs. Understanding the molecular mechanisms of culture-adaptive mutations will facilitate future studies on HCV-host interactions and the development of HCV infectious cell culture systems.

The p7 protein forms oligomers with ion-/proton channel activity^36^, contributing to virion infectivity at the post-assembly step^37^. p7 was found to be essential for infectivity and may interact with other genomic regions in a genotype-specific manner. The amino- and/or carboxyl-terminal intraluminal tails of p7 contain sequences with genotype-specific functions^38^. However, specific regions potentially involved in interactions with p7 were not pursued in the study. Here, by replacing the JFH1 NS4A sequence of p7-J6/JFH1-4A with another genotype 2a (strain J6) sequence or simultaneously replacing both p7 and NS4A into genotype 2b (J8 strain) sequences, we observed increased virus production (Fig. 1). Virus production was affected by introducing F772S and/or A1663V and A1672S, thus providing a direct evidence for the genotype-specific cooperation of p7 and NS4A. As nonstructural proteins from NS3 to NS5B are sufficient for HCV RNA replication^10^ and p7 plays a role in steps of virus replication and assembly^12^, it is likely that the genotype-specific cooperation effects of p7 and NS4A were attributed to the better compatibility of nonstructural proteins in the replication complex and assembly machinery. Nevertheless, no direct physical interactions between p7 and NS4A proteins were identified here in the transient co-expressions. We also demonstrated that the attenuation of NS4A was mediated, at least partially, by J6-4A at RNA level other than the protein level (Fig. 5b–g). Therefore, these data also suggest that the cooperation of p7 and NS4A involves viral RNAs, which warrant further investigations.

p7 substitutions have been identified in several studies and have been shown to increase the infectivity titers of the JFH1 strain^39–42^. F772S was identified in several infectious HCV cell culture systems^23,24,33^ and proven to enhance virus replication. F772S was mapped within the first transmembrane domain of p7 (ref.^9^) and was critical for the translocation of viral proteins into the ER membrane. An alanine-scanning assay suggested that F772S enhances the interaction of p7 and Core protein^33^. However, F772W was found to decrease the infectivity titer of J6/JFH1, and F772 might have multiple interactions within p7 and play a role in stabilizing the protein^43^. The co-presence of F772S and V1663A was also identified in several infectious culture systems of J6/JFH1 with genotypes 1–6 NS4A^23^, indicating the importance of the co-presence of S772 and A1663 for virus production.

Our previous studies found that F772S was important for the viability of J6-JFH1 recombinants and J6 full-length clones^24^. A1672S was recently found to rescue virus replication and assembly defects caused by other NS4A mutations by strengthening the dimerization of the transmembrane domain^44^. However, we found that A1672 was required for the viability of the viruses, since introduction of A1672S or A1663V/A1672S attenuated the J6/JFH1-EGFPΔ40-based recombinants; In addition, F772S-enhanced cooperation of p7-NS4A required A1663 and A1672 (Fig. 3b–f; Fig. 5b–g). These discrepancies might be difficult to explain with a common mechanism because the interactions among HCV RNAs, proteins, and RNA-proteins in the complete HCV life cycle remain largely unknown. It is a possibility that the difference in the genome organization of J6cc and the J6-JFH1 chimeras caused the discrepancies. This does not exclude that the attenuation effect of A1672S here might be attributed to the lack of other adaptive mutations, e.g., those identified together with A1672S in the J6cc^24^. It is highly possible that A1672S work together with the L and G mutations, as LSG mutations had been the foundation for the culture adaptation of full-length infectious HCV clones of genotypes 1a, 2a, and 2b, as well as the 5′UTR-NS5A recombinants of 1–6 genotypes^24–27,45,46^. Our data showed that A1663 was required for F772S-enhanced p7-NS4A cooperation; this is in line with the previous study, in which the co-presence of F772S and V1663A was required for culture adaptation of J6/JFH1-based NS4A recombinants^23^.

Although it was reported that p7 was not required for viral RNA replication^10^, it is indispensable for viral assembly, release and the production of infectious virions^11–13^. p7 was found to be involved in the interaction with NS2, which is crucial for the production of infectious HCV particles^47^. Along with NS5B, it has been shown to promote viral assembly^48,49^ and is required for colocalization with the core proteins, E2, NS2, NS3, and NS5A^37,50^. NS4A functions as a cofactor for the NS3 protease in completing the chymotrypsin-like folding of NS3 and cleaving MAVS to regulate the innate immune response^17,19^. Moreover, p7 and NS3/4A are components of the NS2 complex and are responsible for HCV particle assembly^51,52^. Our study provides direct evidence for isolate-specific p7-NS4A cooperation important for viral assembly and its association with lipid metabolism.

LDs are recognized as organelles that are essential for HCV particle production, mainly because HCV core proteins recruit nonstructural proteins (NS3 and NS4A) and replication complexes (NS4B, NS5A, and NS5B) to lipid droplet-associated membranes^35^. p7 and NS2 have been shown to be determinants in governing the subcellular localization of the HCV Core to LDs and the ER, which is essential for the initiation of the early stage of virus assembly^39^. We found that F772S rescued the size and numbers of LDs that decreased by NS4A replacement (Fig. 6). Moreover, it has been reported that HCV activates NLRP3 inflammasome, which then activates SREBP-1 (lipid-producing transcription factor)^53^, and p7 plays an important role in the activation of NLRP3 (ref.^54^). Our data showed that the difference in the enlargement of LDs was not due to the difference or presence of HCV protein levels but was due to the F772S-enhanced p7-NS4A cooperation (Fig. 6c–h). Recently, another adaptive mutation, C762Y in p7, was found to increase the size of LDs in JFH1-infected cells^48^. Thus, although they were identified in different HCV genomes, the adaptive p7 mutations F772S and C762Y may share a common mechanism for increasing the size and number of LDs, likely also through the p7-NS4A cooperation. Therefore, our data demonstrate that F772S played a primary role in increasing the size of LDs in cells infected with the HCV recombinant with genotype-specific p7 and NS4A.

In conclusion, our study provided the first evidence of the isolate-specific cooperation of p7 and NS4A and the mode of action of adaptive mutation F772S in the complete HCV life cycle. Taken together, these data suggest that p7 coordinates with NS4A to regulate lipid synthesis for efficient virus RNA replication, viral assembly, and release. Small LDs polymerized to form a large droplet to facilitate viral replication and the assembly of infectious particles.

## Materials and methods

### Cell lines

Huh7.5 cells were provided by Dr. Charlie Rice (Apath, L.L.C and Rockefeller University) and maintained in Dulbecco's Modified Eagle Medium (Life Technologies, USA) supplemented with 10% fetal bovine serum (FBS), 100 U/ml of penicillin, and 100 μg/ml of streptomycin (HyClone, USA) at 37 °C with 5% CO_2_.

### Antibodies

Primary antibodies were anti-HCV Core C7-50 (Santa Cruz Biotechnology, USA), anti-HA (MBL, Japan), anti-Flag (MBL), and anti-β-actin-HRP (horseradish peroxidase) (Protein Tech, China). The secondary antibodies were goat anti-rabbit IgG-HRP (Protein Tech) and goat anti-mouse IgG-HRP (Protein Tech) or anti-mouse IgG-Alexa Fluors (Life Technologies). ECL^TM^ anti-mouse IgG and a HRP-linked whole antibody (GE Healthcare, UK) were used for the FFU assay.

### Plasmids

pJ6/JFH1-EGFP△40 contains J6 Core-NS2 and JFH1 5′UTR/NS3-3′UTR, with enhanced green fluorescent protein (EGFP) insertion in NS5A domain III and a 40-aa-deletion (△40) in domain II (ref.^34^), was used as backbone plasmid for cloning. It was designated J6-p7/JFH1-4A in this manuscript, according to the strain origin of the p7 and NS4A sequences. All plasmids were confirmed by sequencing. The primers used in this study are listed in Supplementary Table 1. Transfection of plasmids was performed using Lipofectamine 2000 (Life Technologies).

### Transcription and RNA transfection

Ten micrograms of HCV plasmids were used for linearization, in vitro transcription, and RNA transfection by following the procedures described previously^24,26,55^. The transfected cultures were left for ~16 h and sub-cultured every 2–3 days. The supernatant was collected, filtered (0.45 μm), and stored at −80 °C.

### Determination of HCV infection, FFUs, and sequence*s*

HCV infection in the culture were determined by immunostaining of anti-Core C7-50 or NS5A-EGFP expression, as previously described^24,26,34^. HCV infectivity titers were determined by the FFU assay^24,26,34^. Sequence analysis of the recovered HCV was performed according to the procedures previously described^24,56^, using primers listed in Supplementary Table 1.

### Quantification of intracellular and extracellular HCV RNA

The cells and supernatant were collected at time points as indicated after transfection of equal amount of RNA. J6/JFH1-EGFP△40-GND was the replication-deficient control. Total intracellular RNA was extracted using TRIzol (Life Technologies), and specific RNA was quantitated by real-time RT-PCR (Supplementary Table 1). The PCR results were analyzed using the equation 2^-ΔΔCq^ = (Cq_Target_-Cq_Actin_)_Sample/Xhour_−(Cq_Target_−Cq_Actin_)_Control/4 h_ to obtain the fold-change in expression via the 2^-ΔΔCq^ method. The extracellular level of HCV RNA was quantitated using the COBAS AmpliPrep system^57^.

### SDS-PAGE and western blotting

Total protein was separated on sodium dodecyl sulfate polyacrylamide gel electrophoresis (SDS-PAGE) (60–100 μg per lane) and transferred to a polyvinylidene difluoride membrane (0.2 μm) (Bio-Rad). After transfer, the membrane was blocked (5% milk, 1 h), incubated with primary antibodies (4 °C, overnight), and secondary antibody (room temperature, 2 h). Proteins were visualized with ECL reagents (Protein Tech) and OPTIMAX X-ray Film Processor (PROTEC GmbH, Germany).

### Immunofluorescence confocal microscopy

Cells were fixed by 4% paraformaldehyde in phosphate-buffered saline (PBS) for 30 min at room temperature, permeabilized with 0.2% TritonX-100 in PBS for 10 min, and blocked with 3% BSA in PBS. Slides were stained with anti-Core C7-50 (1:200 dilution) at 4 °C overnight, washed three times with PBS, and stained with IgG-Alexa Fluor-488 (1:250 dilution) (Life Technologies) at room temperature for 2 h. After washing three times with PBS, Oil Red O (Sigma, USA) staining was performed as previously described^58^. The slides were mounted by Prolong Antifade (Life Technologies). Images were collected using a confocal microscope (Zeiss, LSM710) and processed using Adobe-Photoshop-CS5 software.

### Statistical analysis

GraphPad Prism software was used to make graphs (GraphPad Software), and mean ±SEM (the standard deviations of the mean) (*n* = 3 or as indicated) was determined. Student’s unpaired *t* test was used for statistical analysis, and statistical significance was indicated by asterisk(s) (*, *p* ≤ 0.05; **, *p* ≤ 0.01; ***, *p* ≤ 0.001).

### Data availability

We have constructed a number of plasmids in the present study, and the request of plasmids should be addressed to: Yi-Ping Li (lyiping@mail.sysu.edu.cn).

## Electronic supplementary material

Supplementary Table S1
