# Supplementary material for: Adaptive mutation F772S-enhanced p7-NS4A cooperation facilitates the assembly and release of hepatitis C virus and is associated with lipid droplet enlargement
Source: Emerg Microbes Infect. 2018 Aug 8;7:143. doi: 10.1038/s41426-018-0140-z (PMC6081454; doi:10.1038/s41426-018-0140-z)
Supplement: Supplementary file 1 — Supplementary Table S1 [file 41426_2018_140_MOESM1_ESM.docx]

Supplementary Material

**Adaptive mutation F772S-enhanced p7-NS4A cooperation facilitates the assembly and release of hepatitis C virus and is associated with lipid droplet enlargement**

Xiaobing Duan^1^, Muhammad Ikram Anwar^1^, Zhanxue Xu^1^, Ling Ma^1^, Guosheng Yuan^3^, Yiyi Chen^1^, Xi Liu^2^, Jinyu Xia^2^, Yuanping Zhou^3^, and Yi-Ping Li^1, 4, *^

^1^Institute of Human Virology and Zhongshan School of Medicine, Sun Yat-Sen University, Guangzhou 501180, China; Key Laboratory of Tropical Disease Control of Ministry of Education, Sun Yat-Sen University, Guangzhou 501180, China; and Guangdong Engineering Research Center for Antimicrobial Agent and Immunotechnology, Sun Yat-Sen University, Guangzhou 510080, China.

^2^Department of Infectious Diseases, The Fifth Affiliated Hospital of Sun Yat-sen University, Zhuhai 519000, China.

^3^Department of Infectious Diseases, Nanfang Hospital, Southern Medical University, Guangzhou 510515, China.

^4^Program in Pathobiology, The Fifth Affiliated Hospital and Zhongshan School of Medicine, Sun Yat-sen University, Zhuhai 519000, China

*Correspondence should be addressed to: Yi-Ping Li, Research Building, Zhongshan School of Medicine, Sun Yat-sen University North Campus, No.74, Zhongshan 2^nd^ Road, Yuexiu, Guangzhou, Telephone and Fax: (86)20-87335085; E-mail: [lyiping@mail.sysu.edu.cn](mailto:lyiping@mail.sysu.edu.cn)

# Supplementary Table 1. The primers for construction of recombinants and sequencing analysis

| **primer-name** | **sequence** | **The function of the primer** |
| --- | --- | --- |
| J8cc-p7-F | TGTTGGGCCAGGCCGAAGCAGCGCTTGAGAAGCTCATCAT | Amplification of J8-p7 |
| J8cc-p7-R | GCCATGCACAGATGCGTCATAGGCATAAGCCTGCTGTGGTAAGGCCAGGACTAGG |  |
| J8cc-J6 p7-F | ACCACAGCAGGCTTATGCCTATGACGCATCTGTGCATGGC | Fusion J8-p7 into J6/JFH1-EGFP |
| J8cc-J6 p7-R | ATGATGAGCTTCTCAAGCGCTGCTTCGGCCTGGCCCAACA |  |
| J8cc-NS4A-F | AAGCTGACCTTGAGGTCATGACCAGCTCATGGGTCCTGGC | Amplification of J8-NS4A |
| J8cc-NS4A-R | GATGAGAGCCGCCCTAGAGGCGCATTCTTCCATCTCATCAA |  |
| J8cc-JFH1 NS4A-F | TGATGAGATGGAAGAATGCGCCTCTAGGGCGGCTCTCATCGAAGAGGGGCAGCGGATAG | Fusion J8-NS4A into J6/JFH1-EGFP |
| J8cc-JFH1 NS4A-R | CAGGACCCATGAGCTGGTCATGACCTCAAGGTCAGCTTGCATGCATGTGGCGATGTACT |  |
| JF1103 | ACGCAGGGCTTGCGGACGCA | Sequencing the plasmids and virus |
| JF1805 | TGGCACTACCCACCAAGGCA |  |
| JF2487 | ACATCGTCCGATGGGAGTGG |  |
| HF3221 | TCGGATTGGGCTGCTAGTGG |  |
| JF3642 | TCACGCAGATGTACTCGAGT |  |
| JF4273 | CGGCGCCTATGACATCATCA |  |
| JF4871 | CGAGCCTCAGGAATGTTTGA |  |
| JF5470 | TGAGATGGAAGAGTGTGCCT |  |
| JF6091 | CCAATGGATGAACAGGCTTA |  |
| JF6691 | TTGCCAACTACCTTCTCCAG |  |
| JF1241 (KpnI) | GTCCAAGACTGCAATTGCTC | Amplicon including KpnI (1276) and AvrII (3867) |
| JR3900 (AvRII) | GCTGCTCGGAAGAGCCCAAC |  |
| JF3611 (SpeI ) | AACAAGACTCTAGCCGGCTT | Amplicon including SpeI (4106) and RsrII (7341) |
| JR7491 (RsrII) | CGCTGAACTTGTGGCCGTTTA |  |
| J6cc-NS4A-F | AAGCTGACCTTGAGGTCATGACCAGCACATGGGTCTTGGCAGGG | Amplification of J6cc-NS4A |
| J6cc-NS4A-R | GCCGCCCTAGAGGCACATTCCTCCATCTCATCAAAAGCCTCATA |  |
| JFH1-J6cc(NS4A)-F | TGATGAGATGGAGGAATGTGCCTCTAGGGCGGCTCTCATCGAAGAGGG | Fusion J6-NS4A into J6/JFH1-EGFP |
| JFH1-J6cc(NS4A)-R | TCCCCCTGCCAAGACCCATGTGCTGGTCATGACCTCAAGGTCAGCTTGCATG |  |
| J6cc-NS4A(S1672A)-F | GCCGTCGCCGCGTATTGCCT | Introducing the mutation S1672A |
| J6cc-NS4A(S1672A)-R | AGGCAATACGCGGCGACGGC |  |
| J6-NS4A（A1663V）-F | GGGTCTTGGTAGGGGGAGTC | Introducing the mutation A1663V |
| J6-NS4A（A1663V）-R | GACTCCCCCTACCAAGACCC |  |
| JF(F772S)-F | TATTTTGTCATCTTTTCCGT | Introducing the mutation F772S |
| JF(F772S)-R | ACGGAAAAGATGACAAAATA |  |
| J6/JFH-R(10044) | CTATGGAGTGTACCTAGTGTGTGC | Reverse transcription |
| J6/JFH-(F40) | CTCCCCTGTGAGGAACTACTGTCTTCACGC | Amplification of HCV full length (the first PCR) |
| J6/JFH-R(10037) | TCGGACTGAAGATGATAGGCTCAA |  |
| J6/JFH-E-F(1201) | AGCCCAAATGTTCATTGTCTCGCC | Amplification of HCV fragment (including p7) |
| J6/JFH-E-R(3264) | TCGGACTGAAGATGATAGGCTCAA |  |
| J6/JFH-E-F(4748) | CTGGACCCCACCTTCACTATAACCAC | Amplification of HCV fragment (including NS4A) |
| J6/JFH-E-R(6786) | AGGAATTAAGCCCAACGCAGAACGAGA |  |
| J6/JFH-E-F(2182) | CTACCCCTACAGGCTCTGGCATTA | Sequencing the HCV fragment (p7) |
| J6/JFH-E-F(5109) | TAGTAGCCTACCAAGCTACGGTGTGC | Sequencing the HCV fragment (NS4A) |
| RT(+RNA) | CTATGGAGTGTACCTAGTGTGTGC | Reverse transcription |
| RT(-RNA) | GATGTATGGATCAGTATACTCCGT | Reverse transcription |
| q-HCV-F | GGATCAGTATACTCCGTGAATCC | Quantification of intracellular HCV RNA |
| q-HCV-R | TCAGTTCGTGGTGAGAGTATG | Quantification of intracellular HCV RNA |
| q-β-actin-F | GCATGGAGTCCTGTGGCA | Intracellular control |
| q-β-actin-R | ACTTGCGGTGCACGATGGAGG | Intracellular control |

All the primers were synthesized in Sangon Company (China) and purified by PAGE. The primers for PCR were diluted in sterilized water. The concentration **of** stock solution **was 100 μm, and the** working concentration **was 10 μm.**

^*^, the primer for reverse transcription was diluted in RNase-free water.
